# Enhancement of IUdR Radiosensitization by Low-Energy Photons Results from Increased and Persistent DNA Damage

**DOI:** 10.1371/journal.pone.0168395

**Published:** 2017-01-03

**Authors:** Emilie Bayart, Frédéric Pouzoulet, Lucie Calmels, Jonathan Dadoun, Fabien Allot, Johann Plagnard, Jean-Luc Ravanat, André Bridier, Marc Denozière, Jean Bourhis, Eric Deutsch

**Affiliations:** 1 INSERM U1030, Gustave Roussy, Université Paris-Saclay, Villejuif, France; 2 Plateforme de Radiothérapie Expérimentale, Département de Recherche Translationnelle, Institut Curie, Orsay, France; 3 Département de Radiothérapie, Gustave Roussy, Université Paris-Saclay, Villejuif, France; 4 CEA, DRT/LIST, Laboratoire National Henri Becquerel, Gif-sur-Yvette cedex, France; 5 Laboratoire des Lésions des Acides Nucléiques, Univ. Grenoble Alpes, INAC-SCIB, Grenoble, France; CEA, INAC-SCIB, Grenoble, France; 6 Department of Oncology, Radiation Oncology Service, Centre Hospitalier Universitaire Vaudois, Lausanne, Switzerland; 7 Faculté de médecine du Kremlin Bicêtre, Université Paris-Saclay, Kremlin Bicêtre, France; Tulane University Health Sciences Center, UNITED STATES

## Abstract

Low-energy X-rays induce Auger cascades by photoelectric absorption in iodine present in the DNA of cells labeled with 5-iodo-2’-deoxyuridine (IUdR). This photoactivation therapy results in enhanced cellular sensitivity to radiation which reaches its maximum with 50 keV photons. Synchrotron core facilities are the only way to generate such monochromatic beams. However, these structures are not adapted for the routine treatment of patients. In this study, we generated two beams emitting photon energy means of 42 and 50 keV respectively, from a conventional 225 kV X-ray source. Viability assays performed after pre-exposure to 10 μM of IUdR for 48h suggest that complex lethal damage is generated after low energy photons irradiation compared to ^137^Cs irradiation (662KeV). To further decipher the molecular mechanisms leading to IUdR-mediated radiosensitization, we analyzed the content of DNA damage-induced foci in two glioblastoma cell lines and showed that the decrease in survival under these conditions was correlated with an increase in the content of DNA damage-induced foci in cell lines. Moreover, the follow-up of repair kinetics of the induced double-strand breaks showed the maximum delay in cells labeled with IUdR and exposed to X-ray irradiation. Thus, there appears to be a direct relationship between the reduction of radiation survival parameters and the production of DNA damage with impaired repair of these breaks. These results further support the clinical potential use of a halogenated pyrimidine analog combined with low-energy X-ray therapy.

## Introduction

Radiation therapy is a cornerstone of cancer management. Most patients who overcome cancer are cured by a loco-regional treatment, in which radiotherapy plays a prominent role. However one third of patient fatalities are linked to loco-regional relapses, indicating that it is absolutely necessary to increase the antitumor efficacy of radiotherapy for these patients. In addition, a decline in treatment-associated side effects (without compromising antitumor efficacy) is needed for patients who are cured by radiotherapy. Strategies to overcome such limitations include methods which allow individual cancer cells to be targeted with precise radiation dose deposition, and one way to achieve this is to combine radiation therapy with chemical compounds. Many of them have been used as radiation sensitizers to increase the therapeutic index of radiotherapy [1]. Among them, molecules labeled with high-Z atoms such as halogenated pyrimidines, platinium salts or gadolinium texaphyrin, have been or are currently being tested in clinical trials [2–8].

Molecules encompassing high-Z atoms have the capacity to increase the energy deposited by radiation. When a low-energy photon interacts with the atoms of the media via a photoelectric effect, it ejects an electron from the inner electron orbital surrounding the atomic nucleus. A subsequent shell electron reorganization cascade produces a localized spray of low-energy X-rays and electrons. They can deposit a dose with greater relative biological effectiveness (RBE) due to a high linear energy transfer (LET) in aqueous solution [9, 10]. These Auger electrons produce a dense distribution of ionizations, similar to alpha particles [11], with one particle having the ability to cause single-strand and double-strand breaks (DSBs) in DNA. This enhanced radiosensitization through the photo-activated Auger electron cascade is also called photoactivation therapy (PAT).

Halogenated pyrimidines are the most widely used high-Z atom labeling compounds and among them, 5-iodo-2’-deoxyuridine (IUdR), which is a thymidine analogue labeled with an iodine atom, has been well studied. The use of a stable iodine atom avoids the drawbacks resulting from IUdR labeled with Auger emitter radioisotopes such as ^125^I, which are toxic to healthy fast growing tissue such as bone marrow. IUdR is incorporated into DNA during the S phase of the cell cycle and specifically targets actively dividing cells and consequently, tumor cells [4]. Five to ten percent DNA substitution can be achieved for rapidly proliferating tumors such as high-grade gliomas or sarcomas [1, 6, 12]. However, although the ability of IUdR to radiosensitize was widely described *in vitro* more than 30 years ago, no significant radiosensitizing effect has been observed *in vivo* and clinical applications were mostly disappointing [6, 7, 13]. A better understanding of the molecular mechanisms leading to the biological effects of IUdR photoactivation is a key step for further PAT development.

The increased effectiveness of external radiation therapy induced by IUdR incorporation partially occurs via mechanisms related to the biochemistry of DNA damage and repair [14–16], corresponding to radiosensitization, and partially through enhancement of the dose directed at DNA from the localized dose created by Auger electrons which corresponds to the photoactivation contribution. This increase depends on the percentage of base replacement and on the initial photon spectrum [4, 6, 17–25]. These results suggested that the major radiosensitization mechanism could be related to the Auger cascade resulting from ionization of the iodine inner shell [19, 21]. The challenge facing us is to determine the best way to use X-rays to improve the energy deposited by ionizing radiation and its biologic effect. In such therapy, the dose is best delivered when photons with energy above the K-edge of the high-Z atoms, i.e. exceeding 33.169 keV as in the case of Iodine, are used. Although radiosensitization by IUdR has been demonstrated *in vitro* with various radiation sources and photon mean energies of up to 15 MV [15, 17–19, 24, 26–30], the maximum theoretical dose enhancement ratio (DER, S1 Supporting Information) is obtained at 50 keV [17, 19]. This was confirmed in cell survival experiments using a monochromatic photon beam produced by a synchrotron [17].

Based on these previous data, two observations were possible. First, IUdR enhances the dose delivered to the DNA (compared to no IUdR) and has a maximum efficacy at close to 50 keV. Second, at photon energies just above the iodine K-edge, damage caused by Auger electron emission combined with the ability of IUdR to destabilize the DNA produces an overall increased sensitization enhancement ratio (SER). Synchrotron core facilities are not adapted for the routine treatment of patients and these structures are too heavy to be democratized. This uncovered medical need stresses the importance of developing new strategies for adapted therapy that is more accessible and less expensive.

To try to circumvent these limitations, we produced pseudo-monochromatic photon beams with mean energies of 42 and 50 kV respectively by applying specific filtration on a conventional polychromatic X-ray beam. The relative biological effect of each pseudo-monochromatic photon beam was analyzed and showed that considerable IUdR radiosensitization could be obtained without any specific filtration. The mechanism of the sensitization resulting from the Auger cascade is not completely understood. DNA destabilization by a halogenated pyrimidine could result from an increase in the initial level of DNA damage [31] or a decrease in the repair of DNA lesions [25, 32]. Nevertheless, no study has clearly proven these hypotheses to be true. In this study, we followed up the production and late repair of DNA damage events and particularly sites of double-strand breaks (DSBs) through 53BP1 and γ-H2AX labeling [33–37]. We showed that IUdR radiosensitization was associated with increased DNA damage per nucleus in which repair was delayed.

## Materials and Methods

### X-ray tube settings

We used a Varian NDI 226 X-ray tube, with a 30° Tungsten anode angle and an inherent filtration of 0.8 mm Beryllium. In standard conditions, a tension of 200 kV and an intensity of 15 mA are applied with an additional filtration of 0.1 mm of Copper (Cu), a maximal field size (22cmx19cm) and a large focus. At a collimator-sample distance (CSD) of 23.5 cm, the dose rate to water is 1.23 Gy/min ± 5% (RX200). Two other settings were used for biological assays: (i) 70 kV and 30 mA with a 0.1 mm thick copper filter and a 46 cm CSD (RX70) and (ii) 70 kV with a 0.4 mm thick copper filter and a 6.5 cm CSD (RX70F). The mean dose rates to water for these two settings were 0.1035 Gy/min ± 5% and 0.1055 Gy/min ± 5.4% respectively (S1 Supporting Information).

### ^137^Cesium gamma beam

The gamma beam (^137^Cs) was produced by an IBL 637 n°9418 device manufactured by CIS BIO (CEA Saclay). It contains four ^137^Cesium sources “CSL 20R” (n° R042-R060-R068-R052) which are gamma photon emitters with a 662 keV mean energy and a total activity of 138.33TBq (3739Ci), as measured on April 20^th^ 2009. In sample irradiation conditions, the measured reference dose rate was 1.37 Gy/min (± 5%) for the January-June 2013 period. The dose rate was decreased by 1% every 6 months.

### Cell culture

The rat glioblastoma F98 cell lines were obtained from ATCC® (CRL-2397™). The human glioblastoma cell line SF763 from the tissue bank of the Brain Tumor Research Center (University of California-San Francisco, San Francisco, CA) was kindly given by Dr M. Dutreix (UMR3347, Institut Curie, France). Cells were cultured and grown as monolayers in plastic tissue culture disposable flasks (TPP) in Dulbecco’s modified Eagle’s minimum medium with Glutamax (Thermo Fisher Scientific), supplemented with 10% fetal calf serum (PAA) and 1% penicillin and streptomycin (Thermo Fisher Scientific). Cells were grown at 37°C in a humidified atmosphere of 5% CO^2^ in air. The doubling times of F98 and SF763 cell lines were about 20 hours.

When IUdR was used, IUdR (TCI) was diluted in fresh medium from a 1 mM solution in PBS extemporaneously prepared. Forty-eight hours before irradiation, cells were incubated with 10 μM IUdR. Medium containing IUdR was refreshed after the firsts 24 hours and replaced by fresh medium without IUdR just before irradiation. 5-iodo-2’-deoxyuridine incorporation and thymidine replacement evaluation are detailed in Figure A in S1 Supporting Information.

### Viability assays

Cells were harvested using Accutase (Merck) and seeded in triplicate at low-density dilution with or without IUdR in white 96-well plates (Nunc) for survival assays. For F98 cells, 100, 200 and 300 cells were seeded while 200, 300 and 400 cells were seeded for SF763 cell line. The following day, the culture medium was replaced by fresh medium supplemented or not with IUdR. Twenty-four hours later, the medium was again replaced by fresh medium without IUdR. The cells were then irradiated with the indicated beams at 2, 3, 4, 5 and 6 Gy. After a 96-hour cell growth period, the medium was removed and replaced by medium at room temperature. An equal amount of CellTiter-Glo® Reagent (Promega) was added to each well and plates were maintained at room temperature for 10 minutes. Then plates were shaken on an orbital shaker for 2 minutes at 60 rpm before being read on a plate reader set in luminescence mode, as recommended by the manufacturer (S1 Supporting Information).

### 53BP1 immunofluorescent staining

Cells were seeded in 96-well plates (TPP). Twenty-four hours later, the culture medium was replaced by fresh medium supplemented or not with IUdR and again the following day to allow a 48-hour incubation with IUdR. Just before irradiation at 4 Gy with the indicated beams, the medium was again replaced by 100 μl of fresh medium without IUdR. The cells were incubated one hour after irradiation at 37°C and then were washed once with 100 μl of 1X PBS and fixed in 50 μl of 4% paraformaldehyde solution (Electron Microscopy Sciences) for 15 min at room temperature. The cells were then washed twice with 1X PBS before incubation for 10 min in 50 μl of permeabilization buffer (0.5% Triton X-100 in 1X PBS). After a 20-min incubation in 100 μl of 1X PBS supplemented with 2% SVF, the cells were incubated for one hour at room temperature with a rabbit polyclonal antibody against 53BP1 diluted in the previous buffer (Bethyl, 1/400 in PBS/SVF, 50 μl per well). The cells were then washed twice with PBS/SVF before incubation in a 50 μl dilution of secondary antibody (Alexafluor 568 goat anti rabbit antibody, Life technologies, 1/300 in PBS/SVF). After a wash with 1X PBS, the cells were incubated in 100 μl of 1X PBS supplemented with DAPI (Life technologies, 0.1μg/ml) for 10 min at room temperature, washed once again with PBS and directly imaged using an EVOS*fl* microscope (AMG) at 40x magnification.

### Automated counting of 53BP1 foci

Foci were automatically counted in each nucleus, using a home-made ImageJ macro.

#### Nuclear image processing

potential uneven illumination was corrected by dividing the original nuclear image by a duplicate convolved by a gaussian filter (radius: of 20 pixels).The resulting image intensities were enhanced using a gamma transform (gamma: 0.5) and smoothed by a median filter (radius: 5 pixels). Automated thresholding was applied, followed by morpho-mathematics operations (close, dilate, fill holes and watershed). The "analyze particle" function was used to isolate individual nuclei and retrieve their contours.

#### Foci counting

the outline of each nucleus was drawn onto the image of DNA repair foci. As foci were characterized as local maxima, the corresponding ImageJ function was used to identify and count them (noise parameter: 10). Eventually, a montage was created, presenting each nucleus with foci overlaid for visual inspection and assessment of the detection.Flow cytometry analyses

Cells were harvested using Accutase (Milipore) and 20,000 cells were seeded in 12-well plates (TPP) in the presence or in the absence of 10 μM IUdR. The following day, the culture medium was replaced by fresh medium supplemented or not with IUdR. Twenty-four hours later, the medium was again replaced by 1mL of fresh medium without IUdR. For γH2AX staining, cells were irradiated at 4 Gy with the indicated beams. Cells were trypsinized 1, 2, 4, 7, 24 and 48 hours after irradiation. Cells were then treated following the instructions in the FlowCellect^TM^ Histone H2AX Phosphorylation Assay kit (Merck). Acquisitions and the analysis were performed using the Guava EasyCyte HT (Merck) cytometer.

#### Data analysis

Data analyses were performed with GraphPad Prism software. The linear quadratic model was applied to fit the survival curves. Depending on experimental design, the two-way ANOVA Sidak, Holm-Sidak or Tukey multiple comparisons tests with α = 0.05 were applied: “*” corresponds to p<0.05, “**” to p<0.001, “***” to p<0.001 and “****” to p<0.0001.

## Results

### A low-energy photon beam dose rate decrease has no impact on IUdR radiosensitization

IUdR-mediated radiosensitization is best achieved in cells exposed to low-energy X-rays and moreover with photons whose energy is close to 50 keV [15, 17–19, 23, 24]. Since synchrotron core facilities are not adapted for the routine treatment of patients, we aimed to produce a pseudo-monochromatic photon beam with mean energies close to the maximum dose enhancement ratio applying specific filtration on a conventional polychromatic X-ray beam. In standard conditions (RX200), our X-ray source delivers a polychromatic X-ray beam with photon energies ranging from 20 to 200 keV and a mean of 72.5 keV. Simulations with commercial softwares allowed to determine two calibrations of RX200 in order to generate pseudo-monochromatic X-ray beams with means energies close to 50 keV (Table A in S1 Supporting Information) and to optimize IUdR radiosensitization resulting from iodine photoactivation. These two new configurations, RX70 and RX70F, were calibrated and characterized through X-rays spectra measurement with a CdTe detector (Figure B in S1 Supporting Information) and the first half-value layers (HVL) (Table B in S1 Supporting Information). RX70 and RX70F beams were found to exhibited mean energies of 42.68 and 50.62 keV respectively (Figure C and Table A in S1 Supporting Information).

**Fig 1:**
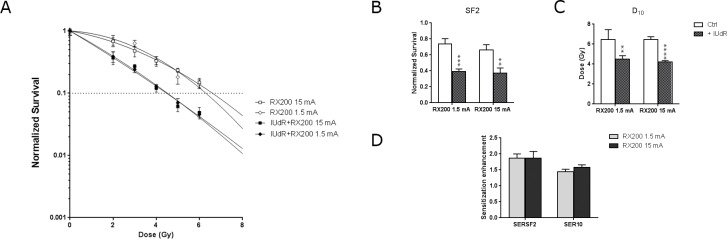
Impact of doserate on IUdR radiosensitization. (A) Cell survival assays obtained with F98 cells incubated in presence or in absence of IUdR before irradiation with RX200 15 mA or RX200 1.5 mA beam. (B) Surviving fractions at 2Gy (SF2), (C) doses giving 10% of cell survival (D_10_) and (D) sensitization enhancement ratios (SER) were determined (n≥3). Complete lists of SF2, D10 and SERs values are available in S1 Supporting Information as Table D and Table E. A polychromatic low-energy X-ray source is sufficient to enhance IUdR radiosensitization.

The new beam calibrations resulted in a 10-fold decrease in the dose rate, from 1.23±5% to 0.1035 and 0.1055±5% Gy/min for RX70 and RX70F conditions respectively (Table C in S1 Supporting Information). To exclude the possibility that these weak dose rates would affect the biological results, we first applied an intensity of 1.5 mA instead of 15 mA in RX200 configuration, leading to a dose rate of 0.1083 Gy/min (Figure D in S1 Supporting Information) and assessed cell viability in cells incubated or not with IUdR before irradiation. We designed a short-time assay based on a miniaturized measurement of the overall cellular ATP content four days after irradiation (Figure E in S1 Supporting Information). As expected for the RX200 configuration (15mA), the cell survival was highly reduced when cells have been pretreated with IUdR (Fig 1A), leading to a great decrease of the survival fraction at 2 Gy (SF2) and the dose required to achieve 10% cell survival (D_10_) (Fig 1B and 1C). In absence of IUdR, SF2 was 0.656±0.060 and became 0.371±0.071 when irradiation was performed after IUdR labeling. Likewise D_10_ values went from 6.359±0.325 Gy without to 4.213 0.111 Gy with IUdR incorporation (Table D in S1 Supporting Information). We next focused on sensitization enhancement ratios at the SF2 level (SER_SF2_) and at the 10% survival level (SER_10_), which correspond to the ratio of the SF2 or the D_10_ for cells without IUdR compared to those with IUdR. Under the 15 mA irradiation condition, SER_SF2_ and SER_10_ were 1.79±0.22 and 1.51±0.06 respectively (Fig 1D and Table E in S1 Supporting Information). Applying a lower intensity (1.5 mA) for irradiation did not alter cell viability results (Fig 1A). Very similar SF2 and D_10_ values were obtained in the absence as well as in the presence of IUdR compared to standard fluency (15mA), yielding 0.735±0.069 and 0.393±0.032 for SF2 and 6.465±0.399 Gy and 4.492±0.140 Gy for D_10_ without or with IUdR incorporation respectively (Fig 1B and 1C and Table D in S1 Supporting Information). As a consequence, the corresponding SER_S_ values were equivalent to those of the RX200 configuration too: 1.87±0.13 and 1.58±0.11 for SER_SF2_ and SER_10_ respectively (Fig 1D and Table E in S1 Supporting Information). These results showed that using photons with energies lower than 200 KeV, applying a weak dose rate had no effect on cell viability at the SF2 and D_10_ levels.

**Fig 2:**
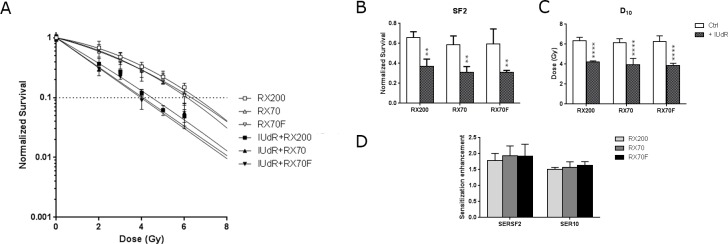
Impact of low energy X-rays on IUdR radiosensitization. (A) Cell survival assays obtained with F98 cells subjected or not to IUdR pretreatment and exposed to the RX200 (72.5 KeV), RX70 (42.5 keV) or RX70F (50 keV) beam. The corresponding (B) surviving fractions at 2Gy (SF2), (C) doses giving 10% of cell survival (D_10_) and (D) sensitization enhancement ratios (SER) were calculated (n≥3).

We next assessed cell viability using the two new beam calibrations, RX70 and RX70F. In the absence of IUdR, the survival curves were similar whatever X-ray beam we used (Fig 2A), showing equivalent SF2 values: 0.656±0.060, 0.588±0.086 and 0.594±0.151, and D_10_ values: 6.359±0.325 Gy, 6.260±0.567 Gy and 6.156±0.385 Gy, for RX200, RX70F and RX70 beams respectively (Fig 2B and 2C and Table D in S1 Supporting Information). As expected, when cells were cultured in the presence of IUdR prior to irradiation, cell viability was reduced, compared to unlabeled cells, for each X-ray beam condition (Fig 2A). Cell viability seemed to be further impaired when the X-ray mean energy of irradiation was close to 50 keV. Indeed, for RX200, SF2 was 0.371±0.071 compared to 0.309±0.059 and 0.310±0.019 for RX70 and RX70F respectively (Fig 2B and Table D in S1 Supporting Information). Likewise D_10_ values went from 4.213±0.111 Gy for RX200 to 3.352±0.619 Gy and 3.851±0.234 Gy for RX70 and RX70F respectively (Fig 2D and Table D in S1 Supporting Information). However, considering the survival enhancement ratios, these weak decreases in cell viability were not statistically significant neither for SER_SF2_ nor for SER_10_. With the standard use of our X ray source RX200, the SER_SF2_ and SER_10_ values were 1.79±0.22 and 1.51±0.06 compared to 1.93±0.31 and 1.92±0.37 for RX70 and RX70F respectively (Fig 2D and Table E in S1 Supporting Information). These results suggested that a conventional low-energy polychromatic X-ray source with a mean close to 50 keV would be sufficient to enhance IUdR radiosensitization.

### A polychromatic low-energy X-ray source yields greater IUdR radiosensitization than ^137^Cesium photons

**Fig 3:**
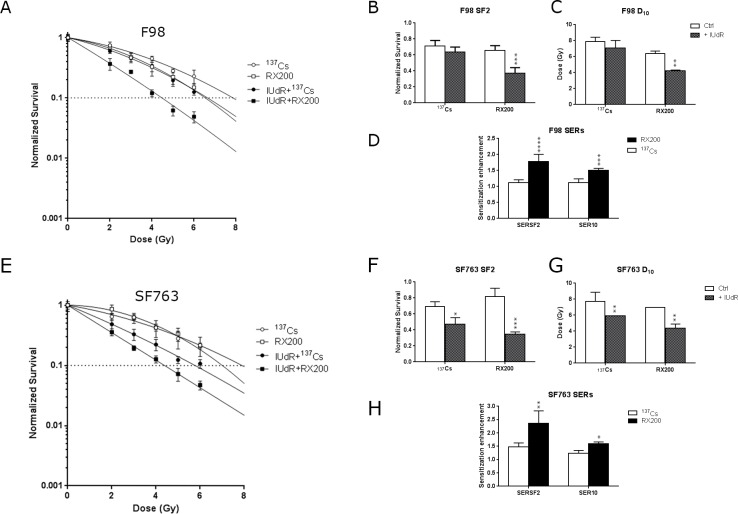
IUdR radiosensitization is greater with with low-energy X-rays compared to ^137^Cesium photons. (A) Cell survival assays obtained with F98 cell line treated with or without IUdR before irradiation with RX200 (72.5 keV) or ^137^Cs (662 keV) (n≥3). Same experiments were performed with human glioblastoma SF763 cells. The corresponding survival assays (D), (E) surviving fractions at 2Gy (SF2), (F) doses giving 10% of cell survival (D_10_) and (G) sensitization enhancement ratios (SER) (G) are presented (n≥3).

We then assessed cell viability after irradiation with the RX200 beam versus a ^137^Cesium gamma beam (^137^Cs). As shown in the Fig 3A, a decrease in cell viability was observed between F98 cells irradiated with RX200 compared to ^137^Cs in absence of IUdR, leading to reduced SF2, 0.656±0.060 compared to 0.712±0.069, and D_10_, 6.359±0.325 Gy compared to 7.868±0.548 Gy (Fig 3B and 3C and Table D in S1 Supporting Information). However, cell viability was further impaired in IUdR treated cells (Fig 3A) and the associated SF2 and D_10_ were highly reduced: 0.371±0.071 compared to 0.640±0.059 and 4.213±0.111 Gy compared to 7.086±0.929 Gy (Fig 3B and 3C and Table D in S1 Supporting Information). Consequently, the corresponding sensitization enhancement ratios were considerably increased from 1.12±0.93 and 1.12±0.12 with ^137^Cs to 1.79±0.22 and 1.51±0.06 with RX200 for SER_SF2_ and SER_10_ respectively (Fig 3D and Table E in S1 Supporting Information). The overall gain in cell radiosensitization using RX200, corresponding to SERs ratios, was 1.59 at the SF2 level and 1.35 at the D_10_ level (Table E in S1 Supporting Information). The same experiments were performed treating SF763, a human glioblastoma cell line (Fig 3E). Very similar results were obtained as for F98 cells for SF2 and D_10_ values (Fig 3F and 3G). The SER_SF2_ and SER_10_ corresponding to SF763 cells irradiated with RX200, 2.36±0.47 and 1.59±0.07 respectively, were greater compared to that obtained with ^137^Cs, 1.48±0.14 and 1.24±0.10 respectively (Table E in S1 Supporting Information). The overall gains in cell radiosensitization using RX200 were 1.61 and 1.28 considering SF2 and D_10_ respectively (Table E in S1 Supporting Information). These observations showed that the decrease in cell viability in the presence of IUdR was mainly due to the photoelectric contribution, since the only difference between the two beams was the photon energy emitted by the source.

### IUdR-mediated radiosensitization is associated with increased DNA damage-induced foci

**Fig 4:**
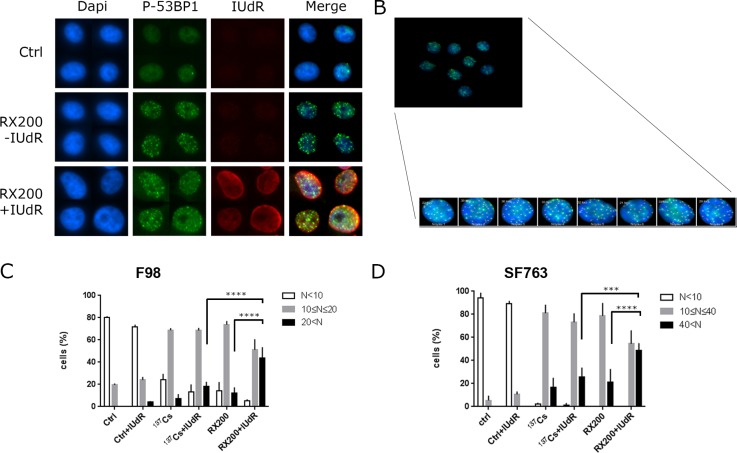
Analysis of DNA double-strand breaks foci. Cells were subjected or not to IUdR pretreatment and then exposed to 4 Gy irradiation with ^137^Cs or RX200 (72.5 keV) conditions. Immunostaining of 53BP1 and IUdR was performed 1 h post irradiation. (A) An example of immunostaining of radiation-induced 53BP1 foci (second column) and IUdR (third column), counterstaining with Dapi (first column) and merge are also shown. (B) An example of automated counting. Range distribution of foci per cell was determined in F98 (C) and in SF763 (D) cells (n = 3).

To further understand the molecular mechanisms associated with IUdR radiosensitization, the number of 53BP1 foci per cell was determined by automated counting after RX200 or ^137^Cs irradiation (Fig 4A and 4B). The analysis of DNA damage-induced foci was performed through their range distribution per cell and demonstrated significant differences. In F98 cells, 43.58±10.0% of IUdR-labeled RX200-irradiated cells exhibited more than 20 foci per nucleus whereas only 12.1±5.3% were detected in RX200-irradiated cells and 18.1±4.6% in IUdR-labeled ^137^Cs-irradiated cells. On the contrary, no significant difference was detected between IUdR-labeled ^137^Cs-irradiated cells and cells irradiated exclusively with ^137^Cs (7.2±4.1%, Fig 4C). Likewise, in human SF763 cells, 48.7±6.3% of IUdR-labeled RX200-irradiated cells exhibited more than 40 foci per nucleus whereas 21.3±11.3% were detected in RX200-irradiated cells, and only 25.5±8.4% in IUdR-labeled ^137^Cs-irradiated cells. No significant difference was detected between IUdR-labeled ^137^Cs-irradiated cells and cells irradiated exclusively with ^137^Cs (16.7±8.4%, Fig 4D). These results demonstrated that the decrease in survival in response to photoactivation of IUdR by low-energy photon irradiation could be correlated with the increased detection of DNA damage-induced foci related to double-strand breaks one hour post irradiation.

### Repair of DNA damage-induced foci is delayed in IUdR-labeled and X-ray irradiated cells

**Fig 5:**
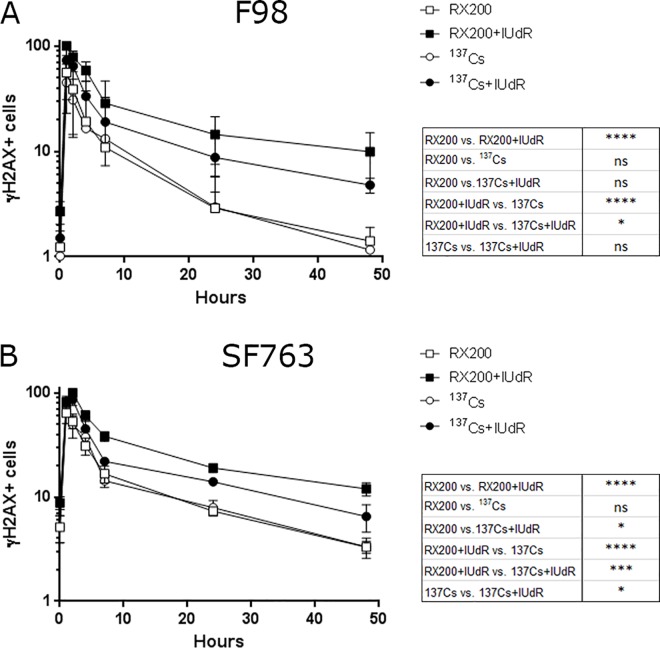
Follow-up of DNA damage trough γH2AX-positive cells. in F98 cells (A) and SF763 (B) cells pretreated or not with IUdR and then exposed to 4 Gy of 137Cs (662 keV) or RX200 (72.5 keV) beam (n≥3). The proportion ofγH2AX-positive cells was represented as a function of the one hour RX200+IUdR condition for F98 cells and the two hour RX200+IUdR condition for the SF763 cell line, where it corresponds to 100%. Statistics corresponding to 48 hours post-irradiation are indicated. The difference to background was significant only for the RX200+IUdR condition in F98 (****) and SF763 (*) cells.

Finally, we followed the DSBs repair kinetics through γH2AX labeling after exposure to IUdR and RX200 or ^137^Cs irradiation. In F98 cells, the maximum proportion of γH2AX-positive cells was reached one hour after irradiation and declined to the basal level 48 hours after irradiation for cells that were only irradiated (Fig 5A). In those cells, the decrease of γH2AX-positive cells was very similar between ^137^Cs-irradiated and RX200-irradiated. As expected, the γH2AX signal in IUdR-labeled irradiated cells decreased slower than that of cells that were only irradiated, irrespective of the irradiation type. Moreover, up to 48 hours post irradiation more γH2AX-positive cells persisted in IUdR-labeled RX200 irradiated cells (9.9±5.1%) compared to IUdR-labeled cells irradiated with ^137^Cs (4.8±0.8%). Similar results were obtained with the SF763 cell line for which the maximum proportion of γH2AX-positive cells was reached two hours after irradiation (Fig 5B). Forty-eight hours after irradiation, 12±1.7% of γH2AX-positive cells were detected in the IUdR-labeled RX200-irradiated cell population compared to 6.5±1.9% among IUdR-labeled ^137^Cs-irradiated cells. As for the F98 cells, no significant difference was observed between cells that were irradiated alone. These experiments indicated that the disappearance of γH2AX-positive cells depends on both the quality of the irradiation and IUdR exposure. In cells which had incorporated IUdR into DNA, repair kinetics were delayed after photon irradiation. Interestingly, for the two cell lines, while 48 hours after irradiation the proportion of γH2AX-positive cells showed significant difference with background for cells that were subjected to IUdR incubation before being irradiated with RX200, it declined to basal level when the combination IUdR+^137^Cs was applied. This last result suggests that the residual γH2AX fluorescence associated with the RX200+IUdR condition 48 hours post irradiation results from unrepaired DNA damages. These findings support the idea that, under these conditions, the DNA damages generated, for which repair takes longer, could be more complex and directly linked to the photoelectric contribution of iodine.

## Discussion

Fairchild *et al*. (1982) were the first to propose the use of stable iodine atoms, with 5-iodo-2’-deoxyuridine (IUdR) incorporated into DNA for photonic activation therapy. As IUdR directly replaces the DNA thymidine base, it is incorporated into DNA during replication in cycling cells [38] and therefore, has the capacity to target tumors exhibiting a high proliferation rate. The radiosensitizing ability of IUdR was demonstrated *in vitro* more than 30 years ago, but clinical applications were mostly disappointing [6, 7, 13, 39].

### X-ray beam characteristics and IUdR radiosensitization

Photon activation therapy proposed by Fairchild and Bond [40], was based not only on the use of IUdR but also on the idea of energetic optimization of the X-ray beam. Previous studies evaluated the dose enhancement ratio (DER, S1 Supporting Information) for various concentrations of IUdR in water. The DER varies with photon energy like a bell-shaped curve with a range of energies yielding a sharp variation from the K-edge of iodine (33 keV) up to 80 keV and reaching its maximum around 50 keV [17, 19, 41]. Monochromatic irradiation of cells exposed to IUdR with a synchrotron X-ray beam over 48 h confirmed that the energy yielding the maximum cytotoxic effect is 50 keV, producing a sensitization enhancement ratio at the 10% survival level (SER_10_) of 2.6 while irradiation at 33.5 keV and 70 keV yielded a SER_10_ of 1.64 and 1.45 respectively in SQ20B cells [17]. However, synchrotron radiotherapy is limited due to the expensive and cumbersome equipment.

Since standard radiotherapy delivered to patients implies the use of megavoltage X-ray sources, to exploit the Auger cascade effect, additional filtrations are required to produce photon beams with sufficiently low energies. The first aim of this study was to produce the energy particles required from a conventional X-ray source. Our X-ray source (RX200) delivers a polychromatic X-ray beam with photon energies ranging from 20 to 200 keV and exhibiting a mean energy of 72.5 keV which is just above the energy yielding the greatest DER. Theoretical calculations validated by physical spectra analysis allowed us to define adapted filtration and energy calibration to generate two pseudo-monochromatic X-ray beams close to the theoretical maximum DER. The mean energies of these X-ray beams were 42 keV (RX70) and 50 keV (RX70F). These new calibrations resulted in a ten-fold decrease in the dose rate. At low dose rate, DNA repair begins while irradiation is still in progress. Consequently, for weak doses some DNA damage had already been repaired before the end of irradiation which is not the case at higher dose rates. To exclude a possible relationship between the low dose rate delivered in the RX70 and RX70F configurations and our previous results, the dose rate of our standard condition of irradiation (RX200) was manually decreased by reducing the amperage applied. As previously described [42, 43], Irradiation with such a reduced dose rate had no impact on cell viability at the SF2 as well as the D_10_ level.

We then compared the radiosensitization enhancement of these three X-ray beams. Cell viability after IUdR exposure was further impaired with the two pseudo-monochromatic beams RX70 and RX70F, compared to RX200 beam. These results are in agreement with those reported by Karnas *et al*. [19] who used the low energies of a polychromatic X-ray beam by operating a 30 keV, a 100 keV and a tungsten filtered 100 keV from a conventional X-ray tube to assess IUdR radiosensitization enhancement. The first two conditions exhibited mean energies below the iodine K-edge whereas the mean energy of the last one was around 50 keV. The greatest enhancement ratios were obtained with the filtered X-ray beam compared to the other two beams (2.3±0.3, 1.8±0.2 and 1.4 for Filtered 100, 100 and 30 keV respectively for a survival level of 1%) and were almost in the same range as those we obtained either at the SF2 or the 10% survival level (from 1.5 to 2.3). The differences we obtained between beams were not statistically relevant. This was probably the consequence of the beam used. Indeed, although we were able to modulate the mean energies by adjusting the applied energy and additional filtration, the beams were still polychromatic and delivered photons for which many energies were common, which is not the case with photons delivered from a synchrotron. However, as the generation of pseudo-monochromatic X-ray beams did not significantly enhance the radiobiological effects of IUdR incorporation into cells, our results showed that the use of an unfiltered orthovoltage beam could be sufficient to exploit photoactivation of IUdR during radiotherapy. Several studies have compared the radiosensitization enhancement produced by low-energy versus high-energy photons produced by X-ray sources. Nath *et al*. [15] studied the survival of Chinese Hamster Ovarian (CHO) cells with and without IUdR for 250 kV and 4 MV X-rays and showed that the SER_10_ was 3.2 and 2.2, respectively. In this case, the gain in the SER_10_ due to the enhanced local dose from the Auger cascade was 1.5. The same year, Miller *et al*. [10] compared radiosensitization of Chinese hamster V79 cells, which had IUdR incorporated into their DNA, with megavoltage (15 MV) versus orthovoltage (100 keV) irradiation. The enhancement ratios at 1% survival levels (SER_1_) obtained with irradiation at 15 MV *vs* 100 keV were 1.8 and 1.95, respectively. This modest 10–15% additional enhancement was much less than that predicted by PAT but is in agreement with those reported by Nath et *al*. [15]. Finally, Dugas *et al*. [18] demonstrated that for IUdR-pretreated CHO cells, the SER_10_ gain is greater with a monochromatic 35 keV photon beam (just above the iodine activation K-edge) than it is with 4 MV X-rays. Here we compared the SER_10_ levels of rat glioblastoma F98 cells and human glioblastoma SF763 cells obtained with the RX200 X-ray beams (20–200 kV) to those obtained with ^137^Cs irradiation (662 keV). We observed that although ^137^Cs was able to radiosensitize cells labeled with IUdR, better radiosensitization was obtained by irradiating cells with RX200. This was reflected by lower SER_10_ levels for ^137^Cs irradiation compared to those obtained with RX200 in F98 cells as well as in SF763 cells: 1.12±0.12 and 1.24±0.1 compared to 1.51±0.06 and 1.59±0.07 respectively. Our results are in good agreement with several other studies in which irradiation was delivered using ^137^Cs or ^60^Co sources [24, 27, 29]. The photon energies emitted by these sources, 662 keV or 1.17 and 1.33 MeV respectively, are much higher than 50 keV. Our results also reflected the IUdR-induced radiosensitization occurring through iodine photo-activation by low-energy X-rays (compared to MeV from radiotherapy irradiators), versus higher energy photons from ^137^Cs irradiation. Indeed, for high energies photons (662 keV), the gain in SER_10_ due to an enhanced local dose deposit linked to photo-electric effect should be weak as it is a minor effect at this energy (only few percent). The SERs ratio values obtained for both cell lines we used were about 1.6 for SF2 and 1.3 for D_10_. These values reflect a gain in SER_s_ that is exclusively dependent on the photo-electric contribution and are totally consistent with those reported by Nath *et al*. [15].

Finally, in all experiments the SER_SF2_ values were greater than that observed for SER_10._ The best gain between ^137^Cs (662 keV), closer to megavoltage used for radiotherapy, and RX200 (72.5 keV), closer to the maximum iodine dose enhancement factor (50keV) was obtained with the survival fraction at 2 Gy (SF2), which is the current gold standard for fractionation radiotherapy protocols. Since the glioblastoma cell lines we worked with were from rat (F98) and human (SF763), these cellular models allow easy transfer to preclinical studies. We do hope that this next step could lead to more promising results.

### DNA damage induced by IUdR photoactivation

Although, many studies have described enhanced IUdR radiosensitization [15, 17–19, 24, 26–28, 30], the molecular mechanisms underlying this phenomenon have not been elucidated. Using a megavolt X-ray beam and a filter elution assay on V79 cells, Kinsella *et al*. [44] showed using elution filters that the production of single-strand breaks and double-strand breaks (DSBs) was increased almost two-fold and 1.5-fold respectively. In our study, we assessed DNA damage through the detection of DSBs foci. In cells labeled with IUdR, a 2-fold increase was detected in those exhibiting a high number of foci when they were irradiated with RX200 compared to those irradiated with ^137^Cs and a 4-fold increase compared to F98 cells only irradiated with RX200. Similar results, but to a lesser extent, 1.9- and 2.3-fold, were obtained with the human cell line SF763. These experimental results confirm that irradiation of cells pre-exposed to iodine with low energy photons has a great potential to increase initial DNA damage. It directly demonstrated the relationship between the decrease in cell viability and the increase in the generation of DNA damage related to iodine photoactivation. Indeed, this increased initial damage reflects an increase of the local dose deposit leading to the associated decrease in cell viability and can explained why a lower dose was required to induce the same survival level

According to the linear quadratic model used to fit the survival curves, the linear component (α) is related to the combination of lesions produced by one particle track to form a lethal lesion [45, 46] and the quadratic component (β) describes the combination of lesions produced by two separate particle tracks to create a lethal event. It was suggested that the Auger cascade resulting from iodine photoactivation produces high-LET particles [9, 10], for which the α component is predominant. This relationship between the increasing dominance of the α component and the dose-response curve leads to the disappearance of the pronounced curvature [15, 17–19, 24]. Considering the loss of the shoulder observed in survival curves, it has been hypothesized that the major effect of IUdR is an increase in the number of single-hit, energy-dense events caused by high-LET-like irradiation [9, 10, 27, 28]. At the molecular level, photoactivation of iodine incorporated into DNA could lead to more complex DNA damage that would exhibit impaired repair kinetics. Our results are in keeping with this hypothesis. Indeed, for the two cell lines we used, more DSBs were still detected up to 48 hours post irradiation when cells were pretreated with IUdR before irradiation with RX200 compared to ^137^Cs and cells treated with irradiation alone. In their study, Kinsella *et al*. [44] did not detect any differences in the repair kinetics of SSBs and DSBs, however this apparent contradiction could be explained by the protocols used. In our experimental conditions, the emitted photons energies were comprised between 20 and 200 keV (mean energy not so far from 50 keV), Kinsella *et al*. used high-energy photons produced by a linear accelerator (15 MV) [44]. Under such conditions, the resulting DNA damage is more likely to reflect IUdR radiosensitization rather than an increase in the local dose deposited resulting from the Auger cascade which occurs with low-energy photon irradiation. The remaining DNA breaks were detected only up to three hours post irradiation. However a lethal lesion will probably be more difficult to repair and remain unrepaired longer. By performing the detection of DNA damage later (at least hours post irradiation), better discrimination of complex, versus other DNA damage, could be achieved. In this manner, we were able to detect significant differences in repair kinetics in cells pre-treated with IUdR and irradiated with RX200. A delay was detected in cells irradiated with ^137^Cs, but it was not significantly different from the background 48 hours after irradiation and was probably the consequence of the “biochemical” destabilization of the DNA structure induced by IUdR incorporation [14–16]. Compared to ^137^Cs, our results showed that with low energy X ray (RX200), DNA repair kinetics was further impaired in presence of IUdR reflecting the presence of residual damages. This increased delay in DNA repair could be explained by the generation of complex DNA damage signature of high-LET particles [47–52]. Moreover since the number of DSBs is increased under such condition, the generation of close DSBs from each other could also lead to the generation of DNA damage more difficult to resolve too. Thus, IUdR photoactivation by low-energy photons is expected to combines the effects of low-LET and of higher-LET particles leading to greater initial DNA damage that is processed slower, that we observed.

Owing to the energy dependence (^137^Cs vs low-energy X-rays) of the sensitization and the DNA damage generated in cells pre-exposed to IUdR, our data suggest that the observed effect is more likely due to the photoelectric effect than to an intrinsic sensitization action of IUdR. We demonstrated here a direct relationship between a decrease in cell survival and increased DNA damage. Moreover, delayed DNA repair was observed in all IUdR-pretreated X-ray-irradiated cells. Our IUdR-mediated radiosensitization is comparable to that obtained with synchrotron core facilities [17, 27]. However, such structures are not adapted for routine patient treatment. Considering the enhanced sensitization observed between megavoltage, classically used for radiotherapy and orthovoltage X-rays, the use of such a beam should be further explored since orthovoltage X-rays are not used in radiotherapy for brain tumors treatment. Although many steps are required to develop new radiotherapy protocols based on the combination of orthovoltage X-rays, IUdR pretreatment and fractionation, our promising results support the interest of such approach for the treatment of aggressive brain tumors such as glioblastomas.

## Supporting Information

S1 Supporting Information (DOCX)
