# Supplementary material for: Enhancement of IUdR Radiosensitization by Low-Energy Photons Results from Increased and Persistent DNA Damage
Source: PLoS One. 2017 Jan 3;12(1):e0168395. doi: 10.1371/journal.pone.0168395 (PMC5207426; doi:10.1371/journal.pone.0168395)
Supplement: S1 Supporting Information — (DOCX) [file pone.0168395.s001.docx]

**5-iodo-2’-deoxyuridine incorporation and Thymidine replacement**

**Materials and Methods**

**Thymidine replacement evaluation**

DNA was extracted and hydrolyzed to corresponding nucleosides using a previously described protocol ([1](#_ENREF_1)). Then, 5-IUdR was detected following HPLC separation using a tandem mass spectrometric detector (HPLC-MS/MS). Detection was accomplished as described for DNA lesions ([2](#_ENREF_2)), in the positive ionization mode using transition 366->239 with a collision energy of 17ev. Thymidine was detected using a UV detector set at 260 nm. Quantitation was performed by external calibration and the results are expressed as the percentage of thymidine replaced by IUdR.

**Flow cytometry analyses**

IUdR labeling was detected using the APC BrdU Flow Kit (BD Pharmigen^TM^), which also recognizes IUdR, following the manufacturer’s instructions. For cell cycle analysis, cells were incubated 30 min in coloration buffer (20µg/ml propidium iodide, 20 mM EDTA, 100 µg/ml RNAse A in 1X PBS). Acquisitions and the analysis were performed using the Guava EasyCyte HT (Merck) cytometer.

**Results**


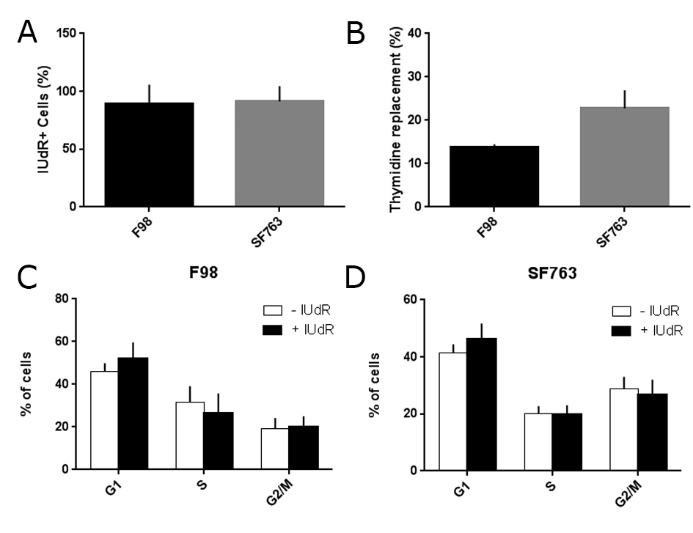
Several studies reported enhancement of 5-iodo-2’-deoxyuridine (IUdR) radiosensitization by low energy photons using different cells lines (V19, CHO, HCT116, HT29, SQ20B or T1 cell lines) and various incubations conditions with IUdR (from 1 to 7 days in presence of 1 to 100 µM IUdR) leading to thymidine replacement ranging from 10 to 20 % of thymidine replacement ([3-11](#_ENREF_3)). In one hand, Nath *et al.* ([9](#_ENREF_9)) showed that over exposition to IUdR leads to cell survival decrease, but in the other hand best radiosensitization enhancements with IUdR are reached when bifilar substitution of DNA is achieved ([5](#_ENREF_5)). The doubling times of F98 and SF763 cell lines were about 20 hours. To ensure that cells where incubated two doubling time with 10 µM IUdR before irradiation, cells were incubated 48 hours in presence of IUdR. Medium containing IUdR was refreshed after the firsts 24 hours. Under these conditions, over 88.2±16.9 % and 91.4±13.2 % of cells (for F98 and SF763 cell lines respectively) were labeled with IUdR (Fig A upper left). The resulting IUdR incorporation yielded thymidine replacement of 13.7±0.6 % 22.8±4.1 % respectively (Fig A upper right). The thymidine replacement used in the present studies had no effect on cell cycle (Figs A lower left and lower right).

Figure A: Cells were incubated 48 h with or without IUdR: (upper left) proportion of IUdR-labeled cells (n=4), (upper right) evaluation of thymidine replacement (n=3) and cycle analysis in F98 (lower left) and SF763 (lower right) cell lines (n=3).

**Production and characterization of pseudo-monochromatic X-rays from a conventional source**

**Materials and Methods**

**CdTe detector, acquisition system and measurement geometry**

X-rays spectra were experimentally determined by the LNHB laboratory using a CdTe detector (XR-100T-CdTe S/N: A015904, Amptek) topped by a tungsten collimator with a small hole (100 µm) to reduce the high flux rate (Fig B). Due to the thickness of the crystal (1 mm) and the small thickness of the beryllium windows (100µm), the intrinsic efficiency values of the CdTe detectors are closed to 1 in the energy range from 20 keV to 50 keV. In our energy range (few keV to 280 keV), the efficiency values provided by the manufacturer were used to correct all the spectra measured. Due to the interaction processes, the measured spectra presented various distortions. Two algorithms were developed by the LNHB to correct spectra, one for the pile-up distortions due to the high count rate, and one for the photon escape phenomenon which takes place into the CdTe crystal. The current intensity was set to 5.5 mA at 70 keV and 1 mA at 200 keV. These settings limited the maximum incoming counting rate on the CdTe detector to 20000 s^-1^.


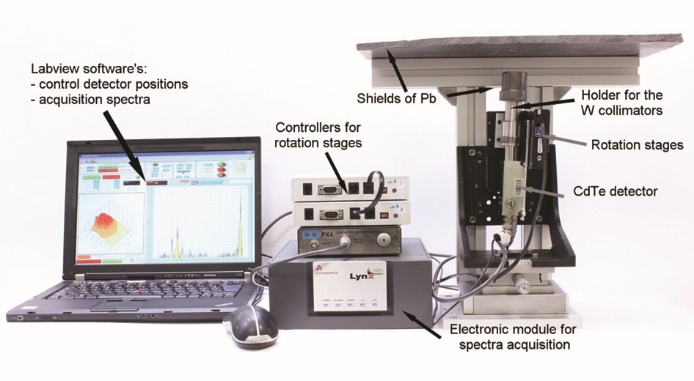


Figure B: Facilities of the CdTe detector for the x-ray beam measurements

The acquisition system coupled to the CdTe detector corresponded to the digital signal processing module LYNX of the Canberra supplier. The settings used are the following: (i) shaping time: Rise time: 0.8 µs, flat top: 0.2 µs, (ii) channel number: 4096 and (iii) energy calibration using two radionuclide standards: ^152^Eu and ^241^Am. The shaping time chosen allowed increase the throughput of the acquisition system for high count rates.

To decrease the incoming counting rate, a group of several tungsten collimators was positioned in front of the beryllium window of the CdTe detector. The different parts of this device were the following: (i) 9 W collimators, thickness: 2 mm, external diameter: 16 mm, hole diameter: 500 µm, (ii) 4 W collimators, thickness: 1 mm, external diameter: 16 mm, hole diameter: 150 µm, and (iii) 1 W collimator, thickness: 2 mm, external diameter: 16 mm, hole diameter: 100 µm. A lead shield of 1 cm thickness, with a hole of 1 cm diameter, was fixed between the detector and the x-ray tube to protect the detector against scattered photons coming from the ground and surrounding structures (Fig B). The distance between the X-ray tube and the beryllium window of the CdTe detector was 1 m.

To obtain a good alignment with the beam axis, a specific device, including two automatic rotation stages, was built. A dedicated Labview^®^ program was developed to control the detector position, research the position corresponding to the maximum of photon emission and acquire spectra automatically.

**Commercial software for X-ray tube beam calculation**

The two commercial software used to calculate the x-ray spectra were XCOMP5 ([12](#_ENREF_12)) and SpekCalc V1.0 ([13](#_ENREF_13)). XCOMP5 cannot take into account a voltage higher than 150 keV. Thus, calculations at 200 keV were carried out using only the SpekCalc V1.0 program. The parameters used to calculate the emitted spectra of the X-ray tube are: high voltage: 70 keV or 200 keV, anode angle: 30°, beryllium window thickness: 0.8 mm, distance between x-ray window and CdTe detector: 1 m. Absorptions inside the different filters which take place between the x-ray tube and the CdTe detector were taken into account (evaporated tungsten layer: 500 nm, barium layer: 22.3 µm, inherent aluminum filter: 1 mm, external copper filter: 0 mm or 0.1 mm or 0.4 mm). The thicknesses of the evaporated tungsten layer and of the inherent aluminum layer were chosen to obtain a good agreement between the calculated and the measured spectra in the low-energy region.

**Half value layer (HLV)**

The condition of narrow beam was necessary. Thus an additional lead collimation of 2 cm by 2 cm was used and positioned at mid distance between the source and the detector. Thin slabs of copper (Cu) or aluminium (Al) were centered on the beam axis and positioned on the additional lead collimator. The detector used was a 0.2 cc ionisation chamber, (NE2577 n°240), which was connected to an electrometer Keithley 35040 under a voltage of 300 Volts. Two measurements of 2 minutes were performed for each metallic slabs added and the first measurement with no slab in the beam was repeated at the end of the HVL experiment. The HVL values were determined from the curve relating the air Kerma rate measured by the detector as a function of the metal thickness. The first HVL corresponds to the metal thickness which divides by two the measured air Kerma rate without any slab in the beam.

**Dose rate determination and X-ray beam quality control**

The dose rate determination was achieved in the X-rays beams with two ionisation chambers (a 15 cc) flat chamber Keithley 96035 (+300V) and a 0.6cc cylindrical chamber NE 2571 (-300V). The detectors were positioned at 1 cm depth in the phantom corresponding to the depth at which the biological samples were irradiated. Irradiation time was two minutes.

The absorbed dose rate to water was determined for the 70 keV X-rays beams according to the recommendations of the dosimetry protocol ([14](#_ENREF_14), [15](#_ENREF_15)):

$$\mathop{D_{water}}\limits^{\bullet} =L_{chamber}xk_{tp}x\left(\frac{\mu_{en}}{\rho}\right)_{air}^{water}xN_{k,chamber}xp_{u}$$

With :

- $\mathop{D_{water}}\limits^{\bullet}$ : the absorbed dose rate to water measured by the ionization chamber

- L_chamber_ : the reading of the ionization chamber

- k_tp_ : the correction factor for ambient temperature t and pressure P compared to the reference conditions (t_0_=20°C and P_0_=1013,25hPa)

- $\left(\frac{\mu_{en}}{\rho}\right)_{air}^{water}$ : the ratio of the averaged mass energy-absorption coefficients of water and air averaged over the spectral energy fluence at the reference depth in the phantom.

- N_k,chamber_ : the air Kerma ionisation chamber calibration coefficient for P_0_ and t_0_ for the used radiation quality . The N_k,chamber_  coefficient is provided by the primary standard laboratory LNHB.

- p_u_: perturbation correction factor (air cavity and wall chamber replaced by water).

Quality control was performed with an 0.6 cc NE 2581 ionisation chamber connected to a NE 2620 electrometer (HV = -330 Volts) irradiated on the beam axis during 2 minutes in a PMMA block centred in the field in a reproducible position on a table. Irradiation settings were 200 keV, 15 mA, 0.1 mm Cu additional filtration, maximal field size, SSD of 42cm. After temperature and pressure corrections, the ionisation chamber measurement was compared to the reference measurement (reference temperature and pressure corrected output (TOP value) = 138.2). The same protocol was used to validate the dose rate associated to the following irradiation settings: 200 keV, 1.5 mA, 0.1 mm Cu additional filtration, maximal field size, SSD of 42cm.

**Dose homogeneity in irradiation field**

The dose homogeneity in the field at the level of the irradiated biological samples was determined for the X-rays and the gamma beams (Cesium-137) with Kodak EDR2 films irradiated at doses of 0.3 and 3 Gy respectively. The films were developed in a dark chamber with film processor Dupont Cronex CX130, scanned with a Vidar scanner and analysed by the Omnipro software. Isodoses were normalized compared to the maximum dose in the beam field.

**Dose Enhancement ratio**

The theoretical enhancement ratio (DER) corresponds to the variation of the mass energy-absorption coefficient of the target due to the presence of iodine:


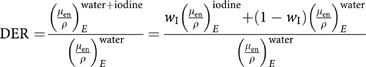


| where | 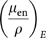 |
| --- | --- |

is the mass energy absorption coefficient for the considered compound irradiated with monochromatic X-ray beam (energy: *E*) and w_I_ is the fraction by weight of iodine in the mixture ([3](#_ENREF_3)).

**Results**


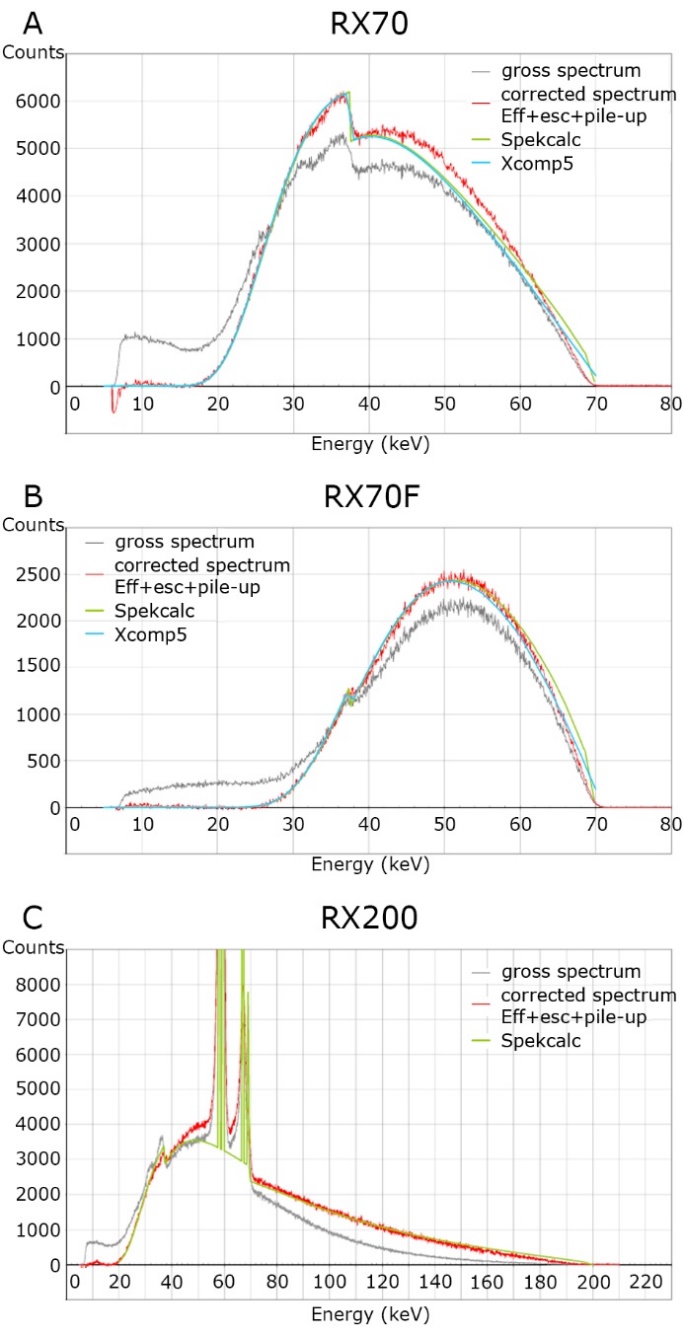
In standard used conditions, our X-ray source delivers a polychromatic X-ray beam with photon energies ranging from 20 to 200 keV, which mean energy above 50 keV, the energy giving the greatest DER for Iodine. The following work focused on the production and characterization of pseudo-monochromatic X-rays beams with a mean energy around 50 keV, and 40 keV, just below the maximum of DER.

| **Configuration** | **Xcomp5 calculation** | **Spekcalc V1.0 calculation** | **CdTe corrected spectra** |
| --- | --- | --- | --- |
| RX70 | 42.81 | 42.48 | 42.68 |
| RX70F | 50.89 | 50.89 | 50.52 |
| RX200 | - | 72.61 | 72.48 |

Table A: Mean energies of spectrum calculated and measured (kV).

Simulations with commercial softwares allowed to determine that a tension of 70 keV combined either with an additional filtration of 0.1 mm of Copper (Cu) or of 0.4 mm Cu should be used to produce X-ray beams with mean energies of close to 40 keV or 50 keV respectively. Since the physical spectrum of the X-ray beam produced in standard used conditions was not known (200 keV, 15 mA, 0.1mm Cu), calculations were also performed for complete characterization of this configuration (table A).X-rays spectra were then experimentally measured with a CdTe detector (Fig B) and compared to the theoretical spectra obtained (Fig C).

The discontinuity observed at 37.4 keV corresponded to the K absorption edge of barium. This element is classically used to trap residual gases inside the X-ray tube. This layer behaves like a thin additional filter between the anode and the CdTe detector. This barium layer had to be taken into account for spectrum calculation by the XCOMP5 or SpekCalc programs. Using the spectra measured with different filtrations at 70 keV, the determination of the barium thickness was possible. The result that we obtained corresponded to 22.3 µm ± 1µm.

Calculated spectra were in good agreement with measured spectra. Some differences could be observed. Indeed, the differences between the minimal and maximal values were equal to 0.8 % and 0.7 % for 70 keV with additional filtration of 0.1 and 0.4 mm Cu (RX70 and RX70F) respectively. For the RX200 configuration, the global shape of the calculated spectrum was in agreement with the measured spectrum. Nevertheless, differences could be observed on the left side of each fluorescence peak of tungsten. These variations came from the well-known phenomenon of charge trapping in the CdTe crystal. This effect produced a tail on the left side of each peak. When the incident energy increased, the tail became significant. Despite this, it was possible to compare the mean energy of these spectra even if the values obtained with the CdTe data could be slightly underestimated. The difference between these values was equal to 0.2 %. These results showed that the deformations of the CdTe spectrum due to the peak tails did not have a strong influence on the final result of the mean energy.

Figure C: Comparison between gross, corrected measured spectra and calculated ones for a 70 kV beam with an additional 0.1 mm-thick copper filter (RX70) (Top), for a 70 kV beam with a copper additional filtration of 0.4 mm thick (RX70F) (middle) and for a 200 kV beam with an additional 0.1 mm-thick copper filter (RX200) (bottom).


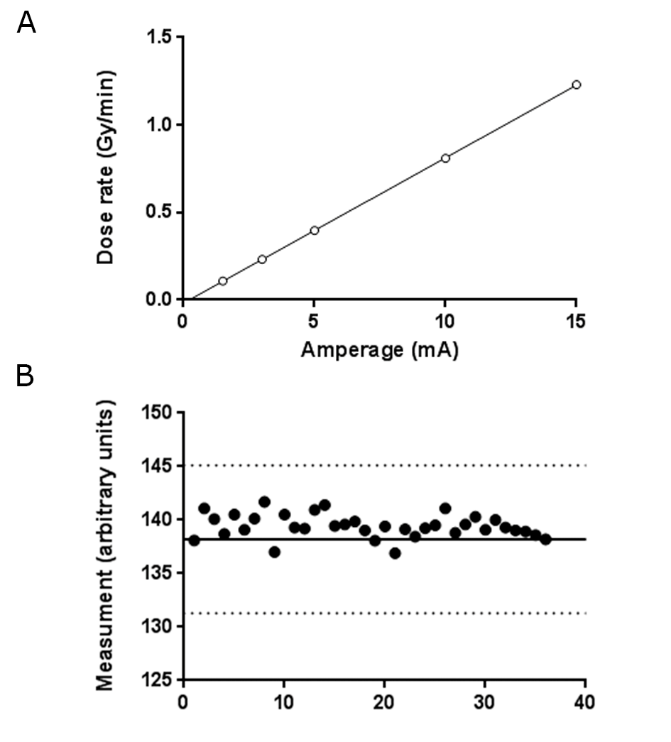
In clinical routine, the X-ray beam quality is also characterized through half-value layers (HVL) and generally by the first HVL and the peak tube potential. The first HLV of a radiation beam correspond to the thickness of an absorber which reduces the air kerma rate of a narrow unidirectional X-ray beam at a point distant from the absorbing layer to 50 % compared to the non-attenuated beam ([14](#_ENREF_14), [15](#_ENREF_15)). The HVL measured were also compared to software’s-predicted values for further validation of X-ray beams. As shown in table B, the measurements confirmed the prediction of the software since the maximum difference was of 4% between the theoretical calculations and the determination from ionisation measurements.

| **Configuration** | **Cylindrical chamber** | **Flat chamber** | **Mean** |
| --- | --- | --- | --- |
| RX70 | 0.103 | 0.104 | 0.1035 |
| RX70F | 0.1044 | 0.1066 | 0.1055 |

Table C: Measured reference dose rates to water for new beam configurations at 70kV and 30 mA (Gy/min).

| **Configuration** | **Xcomp5 calculation** | **Spekcalc V1.0 calculation** | **Measures** |
| --- | --- | --- | --- |
| RX70 | 0.10 mm Cu | 0.11 mm Cu | 2.11 mm Cu |
| RX70F | 5.64 mm Al | 5.81 mm Al | 5.83 mm Al |
| RX200 | 0.47 mm Cu | 0.48 mm Cu | 0.52 mm Cu |

Table B: Predicted and measured HVL values for each beam configuration.

The following step in beam characterization was dosimetry determination. In order to obtained dose rates compatible with biological assays, an intensity of 30 mA was applied and distances to collimator were estimated to obtain equivalent dose rates to water for each new beam configuration. The absorbed dose rate to water in reference conditions of irradiations was measured with two different calibrated ionisation chambers. The mean dose rates at 1 cm depth 70 keV measured with cylindrical and flat chambers were 0.103 and 0.104 Gy/min respectively with 0.1 mm Cu of additional filtration and were 0.1044 and 0.1066 Gy/min respectively with 0.4 mm Cu of additional filtration (Table C). For each configuration, a good agreement (within 2%) was obtained between the two calibrated measuring instruments showing a good traceability of the dosimetric references from the French standard laboratory. Considering ionisation chambers precision, the resulting mean dose rates to water in that conditions were 10-fold lower: 0.1035 Gy/min ± 5% and 0.1055 Gy/min ± 5.4% with additional filtration of 0.1 or 0.4 mm Cu respectively. Since the dose delivered by an X-ray tube is directly proportional to the current intensity and time of insuance, thus by applying an intensity of 1.5 mA instead of 15 mA in RX200 configuration, the doserate of the beam should decrease in a 10-fold manner. We determined that it results in a dose rate of 0.1083 Gy/min (and Fig D Top). The stability of the reference dose rate and the reproducibility of the beam were checked periodically and systematically before each irradiation of biological samples. For acceptability, difference should not exceed ± 5%. During a three years period, only very weak variations were observed and were comprised in a 2% interval demonstrating a great stability of the beam with time (Fig D bottom).

Figure D: (Top) Stability of X-ray beam: TOP values measured during a three years period are plotted on the graph. Dotted lines indicate the ± 5% interval for quality control. (Bottom) Doserate as a function of amperage.

Dose homogeneity in the field was also checked for each X-ray beam configuration and found better than ±10% within the whole surface of the fields used for irradiating the biological samples (data not shown).

**Miniaturized viability assay development**

**Materials and Methods**

**Viability assays**

F98 were harvested using Accutase (Merck) and seeded in triplicate at low-density dilution with or without IUdR in white 96-well plates (Nunc) for survival assays. The following day, cells were then irradiated with RX200 beam at 2, 3, 4, 5 and 6 Gy. After a 96-hour cell growth period, the medium was removed and replaced by medium at room temperature. An equal amount of CellTiter-Glo® Reagent (Promega) was added to each well and plates were maintained at room temperature for 10 minutes. Then plates were shaken on an orbital shaker for 2 minutes at 60 rpm before being read on a plate reader set in luminescence mode, as recommended by the manufacturer.

**Clonogenic assays**


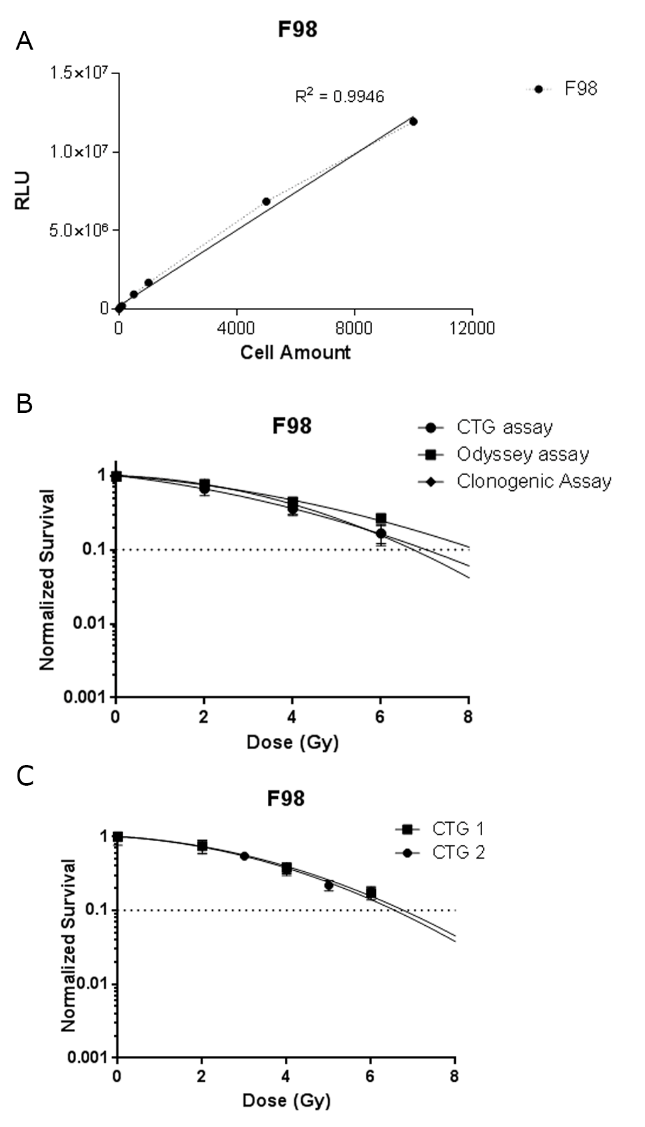
For clonogenic assay, briefly, F98 cells were subjected to 2, 4, or 6 Gy irradiation with RX200 beam. Then cells were harvested and 200, 400 and 800 cells were seeded in 6-well plates and grown for 2 weeks. Cells were finally washed in PBS and subjected to crystal violet coloration. Clones were manually counted.

**Odyssey assay**

For automated nuclei counting, 200, 400 and 800 F98 cells were seeded in triplicates in 12-well. Sixteen hours later, cells were subjected to 2, 4, or 6 Gy irradiation with RX200 beam and grown for one week. Cells were washed twice with 1X PBS before being fixed with 4% paraformaldehyde for 15 minutes at room temperature. Then nuclei were stained with 1.25µM TO-PRO®-3 (Thermo Fischer Scientific) for 10 min (protected from light) at room temperature and automated counted with Odyssey infrared imaging system (Li-COR Biosciences).

**Results**

To make cell viability evaluation easier, we decided to develop, in a 96-well plate format, a more rapid and reliable assay than classical clonogenic assay for our *in vitro* studies on radiosensitivity. The CellTiter-Glo® Luminescent Cell Viability Assay (CTG) is based on the luciferase/luciferin reaction, which in the presence of Mg2+ and ATP, produces oxyluciferin and energy in the form of light. By establishing ATP as the limiting reaction component, this assay measures viability and cytotoxicity of any cell type. We demonstrated experimentally that this reagent is highly sensitive and linear until 10000 cells in 96-well plates (Fig E top). Considering the doubling time of F98 and SF763 cell lines, we design an experiment to evaluate cell viability with the CTG assay in which amounts of cells should not exceed 10000 cells in control wells. 100, 200 and 300 cells F98 cells were seeded in 96-well plates. Cells were submitted to RX200 irradiation with dose ranging from 2 to 6 Gy and were grown in standard conditions four days corresponding to five divisions. Then, using CTG reagent, relative cell viability was determined through ATP amounts. The resulting data were compared with those obtained by performing classical clonogenic assay or by automated cell-counting with Odyssey infrared imaging system (Li-COR Biosciences). As shown in figure E (middle), each method gave very similar results. To ensure that using the CTG assay is a reliable protocol from an experiment to another, we repeated experiments and compared the data obtained. We showed that the survival curves obtained were very similar and even stackable (Fig E bottom). These results underlined the high reproducibility allowed by the use of the CellTiter-Glo® assay for the evaluation of cell viability.

Figure E: CellTiterGlo® (CTG) is a reliable viability assay. (Top) CTG assay sensitivity and linearity. (Middle) Comparison between clonogenic assay, automated nuclei counting assay (Odyssey) and CTG assay performed an F98 cells. (Bottom) Results from two distinct CTG assays are represented.

**References**

1. Ravanat JL, Douki T, Duez P, Gremaud E, Herbert K, Hofer T, et al. Cellular background level of 8-oxo-7,8-dihydro-2'-deoxyguanosine: an isotope based method to evaluate artefactual oxidation of DNA during its extraction and subsequent work-up. Carcinogenesis. 2002 Nov;23(11):1911-8. PubMed PMID: 12419840.

2. Frelon S, Douki T, Ravanat JL, Pouget JP, Tornabene C, Cadet J. High-performance liquid chromatography--tandem mass spectrometry measurement of radiation-induced base damage to isolated and cellular DNA. Chemical research in toxicology. 2000 Oct;13(10):1002-10. PubMed PMID: 11080049.

3. Corde S, Joubert A, Adam JF, Charvet AM, Le Bas JF, Esteve F, et al. Synchrotron radiation-based experimental determination of the optimal energy for cell radiotoxicity enhancement following photoelectric effect on stable iodinated compounds. British journal of cancer. 2004 Aug 2;91(3):544-51. PubMed PMID: 15266326. Pubmed Central PMCID: 2409846.

4. Dugas JP, Varnes ME, Sajo E, Welch CE, Ham K, Hogstrom KR. Dependence of cell survival on iododeoxyuridine concentration in 35-keV photon-activated Auger electron radiotherapy. International journal of radiation oncology, biology, physics. 2011 Jan 1;79(1):255-61. PubMed PMID: 20932663.

5. Fornace AJ, Jr., Dobson PP, Kinsella TJ. Enhancement of radiation damage in cellular DNA following unifilar substitution with iododeoxyuridine. International journal of radiation oncology, biology, physics. 1990 Apr;18(4):873-8. PubMed PMID: 2323975.

6. Karnas SJ, Yu E, McGarry RC, Battista JJ. Optimal photon energies for IUdR K-edge radiosensitization with filtered x-ray and radioisotope sources. Physics in medicine and biology. 1999 Oct;44(10):2537-49. PubMed PMID: 10533927.

7. Laster BH, Thomlinson WC, Fairchild RG. Photon activation of iododeoxyuridine: biological efficacy of Auger electrons. Radiation research. 1993 Feb;133(2):219-24. PubMed PMID: 8438063.

8. Miller EM, Fowler JF, Kinsella TJ. Linear-quadratic analysis of radiosensitization by halogenated pyrimidines. I. Radiosensitization of human colon cancer cells by iododeoxyuridine. Radiation research. 1992 Jul;131(1):81-9. PubMed PMID: 1626052.

9. Nath R, Bongiorni P, Rockwell S. Enhancement of IUdR radiosensitization by low energy photons. International journal of radiation oncology, biology, physics. 1987 Jul;13(7):1071-9. PubMed PMID: 3597150.

10. Sundell-Bergman S, Johanson KJ, Richter S, Ostbergh P. Enhancement of radiation sensitivity in iododeoxyuridine labelled cells exposed to low energy x-rays. Acta oncologica. 1990;29(5):623-6. PubMed PMID: 2206577.

11. Tym R, Todd PW. The Sensitization by Iododeoxyuridine of Cultured Human Cells to the Lethal Effect of X-Rays and Heavy Ions. International journal of radiation biology and related studies in physics, chemistry, and medicine. 1964;8:589-603. PubMed PMID: 14278133.

12. Nowotny R, Hofer A. [Program for calculating diagnostic x-ray spectra]. RoFo : Fortschritte auf dem Gebiete der Rontgenstrahlen und der Nuklearmedizin. 1985 Jun;142(6):685-9. PubMed PMID: 2988070. Ein Programm fur die Berechnung von diagnostischen Rontgenspektren.

13. Poludniowski G, Landry G, DeBlois F, Evans PM, Verhaegen F. SpekCalc: a program to calculate photon spectra from tungsten anode x-ray tubes. Physics in medicine and biology. 2009 Oct 7;54(19):N433-8. PubMed PMID: 19724100.

14. IAEA. Absorbed dose determination in photon and electron beams: an international code of practice,Technical Report Series n°277. Vienna: INTERNATIONAL ATOMIC ENERGY AGENCY; 1987.

15. IAEA. Absorbed dose determination in photon and electron beams: an international code of practice,Technical Report Series n°277. Vienna: INTERNATIONAL ATOMIC ENERGY AGENCY; 1997.

**Table D: SF2 and D_10_ summary**

| **Cell Line** | **Irradiation** | **IUdR** | **SF2** | **D_10_** |
| --- | --- | --- | --- | --- |
| **F98** | ^137^Cs | –  + | 0.71 ± 0.07  0.64 ± 0.06 | 7.87 ± 0.55  7.01 ± 0.93 |
|  | RX200 (15 mA) | –  + | 0.66 ± 0.06  0.37 ± 0.07 | 6.36 ± 0.32  4.21 ± 0.11 |
|  | RX200 1.5 mA | –  + | 0.73 ± 0.07  0.39 ± 0.03 | 6.46 ± 0.40  4.49 ± 0.14 |
|  | RX70 | –  + | 0.59 ± 0.07  0.31 ± 0.07 | 6.16 ± 0.38  3.35 ± 0.62 |
|  | RX70F | –  + | 0.59 ± 0.15  0.31 ± 0.02 | 6.26 ± 0.57  3.85 ± 0.23 |
| **SF763** | ^137^Cs | –  + | 0.69 ± 0.06  0.47 ± 0.82 | 7.75 ± 1.11  5.91 ± 0.01 |
|  | RX200 15 mA | –  + | 0.82 ± 0.10  0.35 ± 0.03 | 6.95 ± 0.03  4.37 ± 0.21 |

Table D: Surviving fractions at 2Gy (SF2) and doses giving 10% of cell survival (D_10_) obtained for F98 and SF763 cell lines treated or not with IUdR and subjected to the various irradiations conditions are summarized.

**Table E: SERs and SERs ratios summary**

| **Cell Line** | **Irradiation** | **SER_SF2_** | **SER_SF2_ Ratios** | **SER_10_** | **SER_10_ Ratios** |
| --- | --- | --- | --- | --- | --- |
| **F98** | ^137^Cs | 1.12 ± 0.93 | – | 1.12 ± 0.12 | – |
|  | RX200 (15 mA) | 1.79 ± 0.22 | 1.59 | 1.51 ± 0.06 | 1.35 |
|  | RX200 1.5 mA | 1.87 ± 0.13 | 1.67 | 1.58 ± 0.11 | 1.41 |
|  | RX70 | 1.93 ± 0.31 | 1.73 | 1.57 ± 0.17 | 1,40 |
|  | RX70F | 1.92 ± 0.37 | 1.71 | 1.63 ± 0.12 | 1,45 |
| **SF763** | ^137^Cs | 1.48 ± 0.14 | – | 1.24 ± 0.10 | – |
|  | RX200 15 mA | 2.36 ± 0.47 | 1.61 | 1.59 ± 0.07 | 1.28 |

Table E: Sensitization enhancement ratios (SER) between cells treated with or without IUdR for the doses 10% of cell survival (SER_10_) were determined for each irradiation condition and both cell lines. SER_SF2_ and SER_10_ ratios for each beam were calculated versus those obtained with ^137^Cs conditions for F98 and SF763 cell lines.
